# Effectiveness of Intraosseous Local Anesthesia (QuickSleeper 5) During Pulpotomy and Stainless Steel Crown Placement on Mandibular Primary Molars: A Crossover Randomized Controlled Clinical Trial

**DOI:** 10.3390/children12030294

**Published:** 2025-02-27

**Authors:** Zeyad A. AlRaddadi, Latifa A. AlHowaish, Ayman M. Sulimany

**Affiliations:** Department of Pediatric Dentistry and Orthodontics, College of Dentistry, King Saud University, Riyadh 11255, Saudi Arabia; lalhowaish@ksu.edu.sa (L.A.A.); asulimany@ksu.edu.sa (A.M.S.)

**Keywords:** pulpotomy, stainless steel crown, effectiveness, local anesthesia, QuickSleeper 5, pediatrics

## Abstract

Background: Effective pain management during dental procedures is essential to ensure positive treatment outcomes, particularly for pediatric patients. Intraosseous anesthesia, administered via the QuickSleeper system, has shown promise as an alternative to traditional local anesthesia techniques. Methods: A single-blinded split-mouth randomized controlled clinical trial took place at the dental hospital at King Saud University with 33 healthy patients (aged 4–9 years), who required pulpotomies and stainless steel crown procedures on two mandibular primary molars, to evaluate the effectiveness of two local anesthetic techniques. Each tooth was randomly assigned to receive 4% articaine either delivered via the intraosseous route using QuickSleeper 5 or buccal infiltration. The effectiveness of the anesthesia was evaluated by the number of injections needed and at various stages using the Sounds, Eyes, and Motor (SEM) scale. Postoperative complications, including pain, swelling, and lip numbness, were assessed through follow-up phone calls with the patients’ legal guardians. Results: The techniques demonstrated comparable effectiveness; there were no statistically significant differences in the number of injections and in the SEM scale scores. Minimal postoperative complications were reported: lip biting (two cases) and prolonged numbness (nine cases) were reported only when buccal infiltration was used, and swelling and pain were reported when both techniques were used. Conclusions: Intraosseous anesthesia via the QuickSleeper 5 system is comparable to traditional buccal infiltration anesthesia for pulpotomies and stainless steel crown procedures in pediatric mandibular molars. Intraosseous anesthesia offers the added benefit of reduced soft tissue numbness and associated complications.

## 1. Introduction

The pain experienced by pediatric dental patients has a considerable effect on treatment outcomes. Hence, it is vital to implement appropriate and timely pain management. This is achieved by controlling painful events and reducing pain and discomfort throughout dental procedures [1]. The American Academy of Pediatric Dentistry (AAPD) [2,3] recommends that pediatric patients be administered adequate local anesthesia (LA) before undergoing any extensive dental procedure, and administering LA serves as the primary approach for alleviating pain associated with dental procedures. However, many individuals delay seeking dental care to avoid LA, and children’s fear of injections during dental procedures is quite apparent [1]. Ideally, LA should have a maximal effect, involve as few injections as possible, cause minimal discomfort, and result in minimal adverse effects [4,5].

In pediatric dentistry, an inferior alveolar nerve block (IANB) and buccal infiltration (BIT) are the main techniques used to induce mandibular LA. The IANB is the technique used most frequently to achieve pulpal anesthesia of the mandibular primary molars, and BIT is the technique most commonly used to administer anesthesia for maxillary teeth. When primary teeth are being anesthetized, it is generally not necessary to advance the needle once it has entered the soft tissues because the apices of the primary teeth are located at the mucobuccal fold [6,7]. In children aged up to five years, BIT is typically adequate to anesthetize the mandibular primary molars [7]; however, the efficacy of infiltration anesthesia for mandibular primary molars is somewhat reduced in older children compared to that in younger children [8].

Recent studies conducted in adult populations have suggested that articaine infiltration may be an alternative to the IANB with lidocaine for the analgesia of the lower teeth [9]. In addition, studies conducted in pediatric populations have shown BIT with articaine to be effective. In two studies designed to evaluate the efficacy of articaine and lidocaine in controlling pain in pediatric populations during pulp treatments of deciduous mandibular second molars, it was found that BIT with 4% articaine produced an anesthetic effect comparable to that of a 2% lidocaine IANB during pulp treatments of mandibular primary molars [10,11]. Furthermore, a meta-analysis showed that the use of articaine rather than lidocaine is more likely to result in successful anesthesia in maxillary and mandibular infiltrations and IANBs for asymptomatic and symptomatic teeth [12].

Administering LA in the oral cavity can have an undesirable clinical consequence: self-inflicted soft tissue injuries. The most common complaint associated with the anesthesia of the mandibular primary molars is that numbness causes patients to accidentally bite their tongue, cheeks, or lips [6]. Although bleeding and infection are possible, most of the resultant lesions are self-limiting and heal without causing further issues [2,3]. The use of BIT has been recommended to reduce soft tissue anesthesia and injuries associated with the use of the IANB [2,8].

According to the AAPD, the intraosseous anesthesia technique (IOT) is reliable in pediatric patients [2]. The advantages of this technique are numerous, and they include an immediate onset of action (30 s), a requirement for a minimal volume of local anesthetic, and a low incidence of postoperative complications [8]. In a review conducted by Tom and Aps [13], it was found that computer-assisted intraosseous anesthesia is a highly successful primary strategy for numbing one or two molars located in the lower jaw and that administering 0.6–0.8 mL of a solution containing 4% articaine and 1:200,000 epinephrine exhibited notable efficacy in young patients, was easily administered, and offered substantial patient comfort. In another study, the QuickSleeper system was found to be effective and reduce the numbness of soft tissues and occurrence of self-biting, suggesting that it could be used in place of, or in addition to, conventional infiltration procedures in children and adolescents [14]. Furthermore, Sixou and Barbosa-Rogier [15] reported that the QuickSleeper 2 system had an overall success rate of 95% (endodontics: 96%, restorations: 100%, and extractions: 88%) for primary teeth treatments. Importantly, no cases of self-biting due to LA were recorded in their study. The IOT reportedly requires less anesthetic solution when compared with other LA techniques, with studies generally indicating that 0.8 mL of anesthetic solution is effective for restorative and endodontic procedures [13,16]. As a result, less epinephrine is delivered, and less local harm is observed when the IOT is utilized compared to other LA techniques [15]. In terms of the heat generated by the QuickSleeper system, Simeonova et al. [17] found that the temperature change that occurred in pig jaws while using the system was minimal and did not harm the bone.

The QuickSleeper 5 (QS5) is a computer-controlled local anesthetic delivery device that is not exclusively an intraosseous device but is also capable of administering local anesthesia through infiltration or IANB, depending on the mode selected by the operator. While some studies have investigated the use of the IOT in pediatric patients, no published studies have compared the effectiveness of LA delivered using the QS5 system with that delivered via BIT. In our previous study [18], we assessed pain-related behavior and pain perception associated with BIT and IOT during the injection of the LA to evaluate which technique is less painful. In this phase of the trial, we focused on evaluating the effectiveness of BIT and IOT within the same study population. Therefore, the aims of this study were to (i) assess the effectiveness of intraosseous anesthesia (4% articaine with 1:100,000 epinephrine) (Septodont, Paris, France) delivered using the QS5 system during pulpotomies and stainless steel crown (SSC) procedures in mandibular primary molars; (ii) compare the effectiveness of using BIT to achieve LA; and (iii) evaluate the postoperative complications associated with each LA technique.

## 2. Materials and Methods

### 2.1. Ethical Approval

This study is part of a larger research project in which intraosseous anesthesia delivered via the QS5 system is being examined in pediatric patients [18]. The study was approved by the Clinical Trial Unit and Institutional Review Board of King Saud University Medical City (registration number: E-22-6879) on 23 August 2022.

### 2.2. Study Design

**Figure 1:**
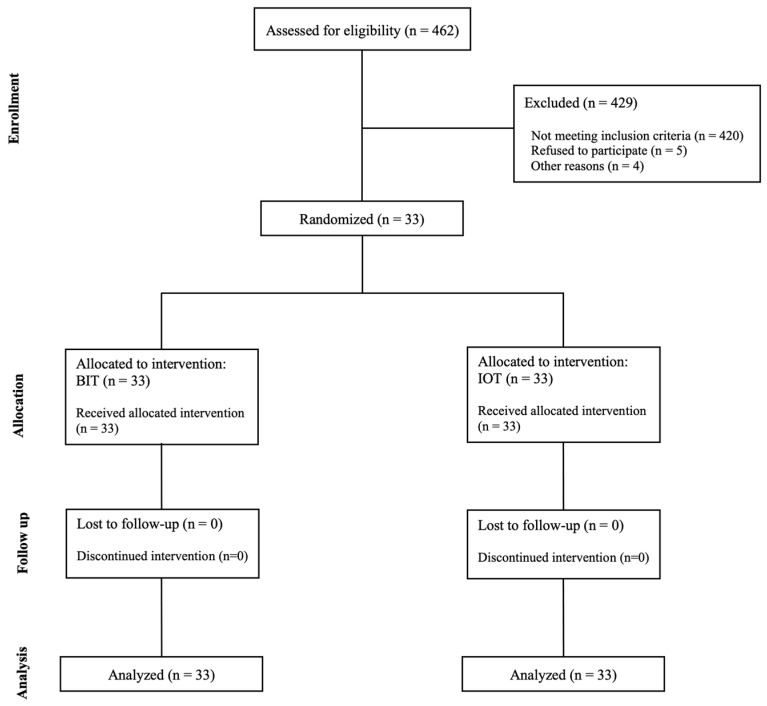
Consort diagram of the sample selection for the two local anesthetic techniques, a traditional technique (BIT) and intraosseous obtained via QuickSleeper 5 (IOT).

In alignment with the CONSORT statement (Figure 1) [18,19], we designed this experiment as a single-blinded split-mouth randomized controlled clinical trial. The study was performed at the Dental University Hospital (DUH) in King Saud University Medical City, Riyadh, Saudi Arabia.

### 2.3. Sample Size Calculation

We determined the sample size using SPSS Version 26.0 (IBM Inc., Chicago, IL, USA). To establish an effect size of 0.6 with 0.90 power at alpha = 0.05, a minimum of 33 patients were required, using effectiveness as the primary outcome for the computation.

### 2.4. Sample Selection

The sample was selected from children who attended pediatric dental clinics at the DUH, had a grade 3 or 4 behavioral rating according to the Frankl Behavior Scale [20], and had two mandibular primary molars indicated for pulpotomy and placement of an SSC. The legal guardians of the potential participants were provided with a thorough explanation of the study, including the risks and benefits of the procedures and the study protocol. Informed consent was obtained from the guardians of all participants.

### 2.5. Randomization

A randomization program (http://www.randomizer.org, accessed on 10 March 2022) was used to generate a randomized list. Each patient received two LA techniques: IOT and BIT. One technique was administered during the first treatment visit, and the other was used during the second treatment visit. A dental assistant kept the randomization list throughout the trial period. Neither the patient nor their legal guardians knew which technique would be given first.

### 2.6. Inclusion and Exclusion Criteria

Children who were four to nine years old, who were healthy with no physical or mental illness, who demonstrated positive or definitely positive behavior, who had healthy periodontal tissue, and who had two lower primary molars indicated for pulpotomy and SSC were included in this study. The patients had no contraindications for administering LA and were required to abstain from using analgesics for at least 24 h before treatment [21,22].

Patients who failed to attend the two treatment visits with a minimum 7-day and maximum 30-day time frame between them were excluded from the study.

### 2.7. Clinical Procedure

#### 2.7.1. Screening Visit

During the screening visit, each patient was screened by the principal investigator, comprehensive medical and dental histories were taken, and clinical and radiographic examinations were performed to formulate a treatment plan. Patients who fulfilled the inclusion criteria were scheduled to attend two treatment visits, and they and their legal guardian(s) were provided with an explanation of the study. Those who agreed to participate were then asked to sign an informed consent form. Patients who did not meet the study’s requirements or who did not agree to participate were referred to the pediatric dentistry department.

#### 2.7.2. Treatment Visits

Before any treatment procedure was initiated, a single operator, who performed both local anesthesia techniques and all the treatment procedures, reviewed the informed consent form, and the legal guardians were provided an opportunity to ask questions. The dental assistant then recorded the treatment details, including the technique to be used, the tooth type, and the number of injections. No pharmacological behavior management techniques were applied; the patient’s behavior was managed with communicative guidance, the tell–show–do technique, the ask–tell–ask technique, positive reinforcement, and nonverbal communication.

#### 2.7.3. Local Anesthesia (LA) Techniques

The LA techniques used in this study have been previously described [18]. Each patient received 1 mL of 4% articaine with 1:100,000 epinephrine at first. However, if the patient experienced pain and required additional local anesthesia, the operator administered the remaining 0.7 mL of the carpule.

#### 2.7.4. Effectiveness of the LA

The effectiveness of the LA was measured using the Sounds, Eyes, and Motor (SEM) scale. This is a 12-item scale, and the final score ranges from 3 to 12 points; lower scores are preferable. The reactions were categorized on a scale from 1 to 4, encompassing four classifications: comfort, mild discomfort, moderately painful, and painful for each of the S, E, and M codes. The sound scale ranges from 1 (absence of sound) to 4 (verbal complaints indicating intense pain, such as screaming or crying). The scale for eye discomfort ranges from 1, indicating no signs of discomfort, to 4, which denotes the patient is crying with tears running down their face. The motor scale ranges from 1, indicating a relaxed hand with no observable body tension, to 4, which signifies movement of the hands aimed at making aggressive physical contact. The pain reaction scores range from a minimum of 3 to a maximum of 12 [23]. The effectiveness was measured during the seven stages of the pulpotomy and SSC placement procedure. The patients were video recorded, and text was added to the video recordings to indicate the seven points at which the effectiveness was to be evaluated: the placement of the rubber dam, drilling, opening of the pulp, removal of pulpal tissue (using a low-speed handpiece), removal of the rubber dam, preparation for the SSC, and placement of the SSC. A single-blinded calibrated independent evaluator who was unaware of the anesthesia procedure assessed and scored the effectiveness of the LA at the seven stages. The data from each stage were used to calculate the mean score for each stage across all patients. Intra-examiner reliability was assessed using Cohen’s kappa.

### 2.8. Blinding

Each patient was video recorded during each treatment procedure with two cameras: one filmed the patient’s body, and the other filmed the patient’s face. This was to capture the patient’s facial expressions and body movements. All the anesthesia instruments were removed prior to starting the recording to ensure that the evaluator was not aware of the LA procedure performed.

### 2.9. Postoperative Complications

Standard postoperative instructions were given to all patients, such as not to bite their lips, and the legal guardians were instructed to observe the patient. In addition, the operator informed the legal guardians that the patient might feel prolonged numbness, and pain might occur after the appointment. Each patient’s guardian(s) was contacted by the operator within 2–4 h of the administration of the LA and asked whether the patient had any postoperative complications and whether numbness was still present. In addition, they were asked about lip biting, swelling, or pain at the injection site.

### 2.10. Data Analysis

Data were analyzed using SPSS 26.0 statistical software (IBM Inc., Chicago, IL, USA). Descriptive statistics (means, standard deviations, frequencies, percentages, and ranges) were used to describe the quantitative and categorical variables. The chi-square test was used to assess the overall effectiveness of both techniques by evaluating the number of injections administered. The paired *t*-test was used to compare the quantitative outcome variables in relation to the categorical study variables (two categories). For the postoperative complications, McNemar’s test was used to detect the difference between the IOT and BIT. When *p* < 0.05, the result was considered statistically significant.

## 3. Results

In this study, we compared the effectiveness of two LA techniques, BIT and the IOT, in the 33 participants. We evaluated the effectiveness via the number of injections administered for BIT and the IOT. In addition, we evaluated the effectiveness of the techniques five times during the pulpotomy (i.e., during the rubber dam placement, tooth preparation, pulp opening, pulpal tissue removal, and rubber dam removal) and two times during the SSC procedure (i.e., during SSC preparation and placement). Table 1 outlines the demographic and clinical characteristics of the participants. Among the 33 participants, 60.6% were four to six years old, and 39.4% were seven to nine years old. The gender distribution data showed that most of the participants were female (60.6% vs. 39.4% male). Most of the teeth treated were of the lower E (60.6% vs. 39.4% lower D). The majority of the participants required a single injection.

Table 2 shows the number of injections administered for each technique for all the patients. Most of the patients did not require additional LA; only six for BIT and nine for the IOT needed a second injection (a total of 1.7 mL 4% articaine with 1:100,000 epinephrine); however, the difference was not statistically significant.

Table 3 shows the mean SEM scale scores associated with the two LA techniques recorded during the five stages of the pulpotomy and the two stages of the SSC procedure. The total scores for each part of the entire procedure are also shown. The mean scores were similar during each stage, with slight variations. The scores did not differ to a statistically significant extent at any stage.

**Figure 2:**
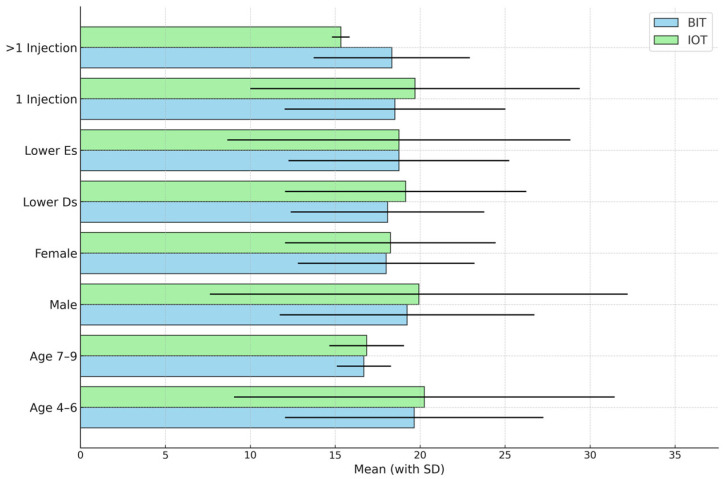
Comparison of the mean Sounds, Eyes, and Motor (SEM) scale scores associated with the two anesthesia techniques recorded during the five pulpotomy stages and according to each of the study variables. The means are the sums of the mean SEM scale scores for the five pulpotomy stages.

Figure 2 compares the mean SEM scale scores recorded during the five pulpotomy stages according to each of the study variables (age, gender, tooth type, and number of injections) using a paired *t*-test. No statistically significant differences were observed.

**Figure 3:**
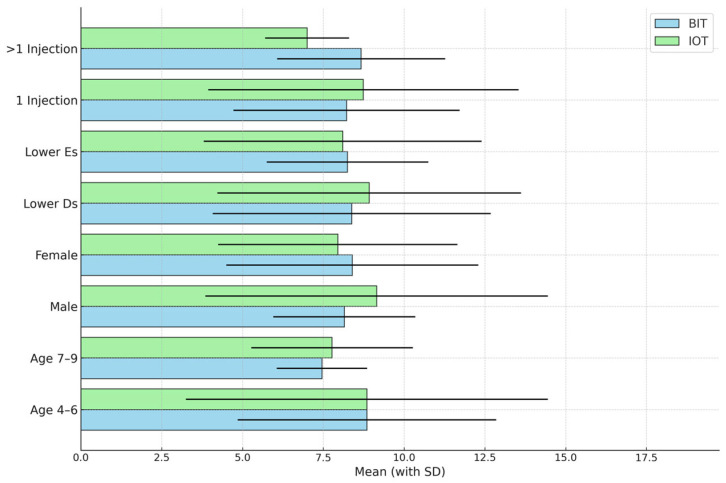
Comparison of the mean Sounds, Eyes, and Motor (SEM) scale scores associated with the two anesthesia techniques recorded during the two stages of stainless steel crown (SSC) tooth preparation and placement of the crown according to each of the study variables. The means are the sums of the mean SEM scale scores for the two stages.

Figure 3 compares the mean SEM scale scores recorded during tooth preparation for the SSC procedure and its placement stages according to each of the study variables (age, gender, tooth type, and number of injections) using a paired *t*-test. No statistically significant differences were observed.

**Figure 4:**
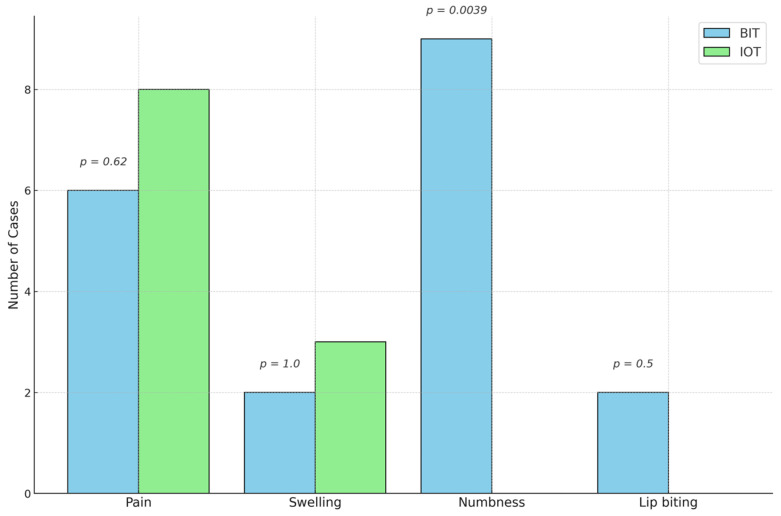
Occurrence of four postoperative complications.

In terms of the postoperative complications experienced by the participants (Figure 4), there were eight cases of pain associated with the IOT and six cases of pain associated with BIT. There were three cases of swelling associated with the IOT and two cases of swelling associated with BIT. Notably, lip biting (two cases) and numbness (nine cases) were associated only with BIT. McNemar’s test showed that there was a significant difference for numbness with a *p*-value of 0.0039.

The intra-examiner agreement was near perfect, as indicated by kappa values > 0.95. A *p*-value of ≤0.05 was used to confirm the statistical significance of the results.

## 4. Discussion

The primary aim of this study was to evaluate and compare the effectiveness of intraosseous anesthesia delivered using the QS5 system with the anesthesia achieved via BIT with 4% articaine for pulpotomies and SSC procedures in the mandibular primary molars of pediatric patients. Additionally, we aimed to assess postoperative complications associated with each of the studied LA techniques. To achieve these aims, we conducted a single-blinded randomized split-mouth controlled clinical trial with 33 pediatric patients (four to nine years old) who required a pulpotomy and SSC procedure on two mandibular molars. Both the IOT and BIT were applied in each patient in a randomized manner across two visits. The effectiveness of the LA was assessed by the number of injections required for each technique and during seven stages of each treatment procedure using the SEM scale, a validated pain assessment tool. The occurrence of postoperative complications (i.e., pain, swelling, lip biting, and numbness) was recorded through follow-up phone calls with each patient’s guardians 2–4 h after each treatment.

Most of the patients did not require additional LA; only six for BIT and nine for the IOT needed a second injection (a total of 1.7 mL of 4% articaine with 1:100,000 epinephrine); however, the difference was not statistically significant. To the best of our knowledge, no studies have evaluated the number of injections in regard to the effectiveness of LA.

The mean SEM scale scores for the IOT and BIT recorded during each of the five pulpotomy stages (placing the rubber dam, drilling, opening the pulp, removing the pulp tissue, and removing the rubber dam) and the two stages of the SSC procedure (preparing the tooth for the SSC procedure and cementing the SSC) were not statistically significantly different (Table 3). The total scores for the two treatment components also did not statistically significantly differ. For the pulpotomy component, the total scores were 18.48 ± 6.1 for BIT and 18.91 ± 8.9 for the IOT (*p* = 0.556). For the SSC placement component, the total scores were 8.30 ± 3.3 for BIT and 8.42 ± 4.4 for the IOT (*p* = 0.875). These results provide further evidence of the equivalency of these techniques in terms of their performance, suggesting that they are comparable.

Our results align with those of Sixou and Barbosa-Rogier [15], who evaluated the effectiveness of intraosseous anesthesia (4% articaine) delivered using the QuickSleeper 2 system in pediatric patients and reported a success rate > 96% for pulpotomy procedures on primary teeth. In addition, our results support those reported by Elbay et al. [24], who evaluated the effectiveness of intraosseous anesthesia against intraligamentary (IL) anesthesia (4% articaine) delivered using the SleeperOne system Dentalhitec (Cholet, France) in patients with a mean age of seven years during various stages of pulpotomy procedures (i.e., drilling in enamel with a high-speed handpiece, drilling the dentin with a low-speed handpiece, and removing the coronal pulpal tissue). Although IL anesthesia was not as effective as intraosseous anesthesia, the difference was not statistically significant. Moreover, the authors concluded that the techniques were equally effective during pulpotomy. In another split-mouth randomized clinical trial, pediatric patients (mean age: seven years) underwent restorations, pulpotomies, or extractions while receiving 2% lidocaine via the QuickSleeper system or an IANB, and the effectiveness of the anesthesia was evaluated [25]. The authors concluded that the IOT and IANB were equally effective; however, they did not mention whether the IOT was more effective for any particular treatment(s). Finally, a split-mouth randomized controlled trial conducted to compare the effectiveness of intraosseous anesthesia delivered using the QS5 system and LA induced by an IANB (2% lidocaine) in pediatric patients aged six to nine years during pulpotomies showed that the IOT was significantly more effective than the IANB, with respective success rates of 92% and 77% [26].

Regarding the effectiveness of the IOT and BIT for inducing anesthesia during the preparation for and placement of an SSC, no statistically significant difference was found in this study. The SEM scale scores for the LA techniques were nearly identical across the two stages, and the similar total SEM scale scores (BIT: 8.30 ± 3.3 vs. IOT: 8.42 ± 4.4, *p* = 0.875) validated the comparability of the two techniques. Thus, our findings suggest that the IOT is comparable to BIT for the delivery of 4% articaine with 1:100,000 epinephrine. We evaluated the effectiveness during the preparation phase, as sometimes there is a need for subgingival procedures. Additionally, we evaluated the placement process to assess the gingiva’s response to the crown’s placement and the associated pressure. There was no statistically significant difference between the two techniques. To our knowledge, the effectiveness of the IOT in pediatric patients during the preparation and placement of SSCs has not been evaluated to date. Therefore, we cannot compare the findings from this part of our study with those from similar studies. It is also not appropriate to compare our findings to those from studies that used other treatments (e.g., extractions or direct restorations) or techniques due to the differing effects that treatments have in terms of patients’ responses to treatments.

When we analyzed the occurrence of four postoperative complications after the administration of the two LA techniques, differences were evident: lip biting (two cases) and prolonged numbness (nine cases) were exclusively reported after BIT. Minor complications (i.e., pain and swelling) occurred after both the IOT and BIT. The only statistically significant difference in the postoperative complications between the two LA techniques pertains to numbness. Our results align with those of Castelo et al. [25], who showed that intraosseous anesthesia delivered using the QuickSleeper system resulted in no postoperative lip biting and significantly fewer postoperative complications than LA administered via the IANB. In another study, which analyzed the effectiveness of the QuickSleeper 2 system, it was found that no lip biting occurred after the delivery of LA (4% articaine) using the IOT, that only slight numbness occurred after the injection, and that no post-injection pain or injury occurred at the site of injection [15]. In contrast, Elbay et al. [24] reported that postoperative complications (namely, pain and swelling) were similar in patients who received IL and intraosseous anesthesia and that lip biting was significantly more frequent in those who received intraosseous anesthesia. This contradicts our findings; in our study, there were no reported cases of lip biting after intraosseous anesthesia. Nevertheless, our findings support the notion that intraosseous anesthesia is advantageous, as it reduces soft tissue numbness and thereby minimizes the risk of self-inflicted injuries, which are common in pediatric patients.

The design of our study presents several limitations. For instance, blinding was not feasible for either the operator or the patients. In addition, it was not feasible to keep the patient waiting after finishing the procedures to evaluate the postoperative complications; therefore, a phone call was made to all the patients’ legal guardians to provide information if any complications occurred. Thus, the collected responses were subjective and may lack standardization. Moreover, we did not ask the legal guardians whether the patient took analgesics after the procedure; it would be unethical to prescribe analgesics for every patient or ask the patients not to take any analgesics. We recommend that future studies incorporate these aspects into their design.

Conversely, some of the strengths of our design must be noted, such as having a single trained operator executing all the procedures, a blinded calibrated evaluator with excellent kappa agreement, and a split-mouth design to mitigate inter-individual variabilities. Furthermore, each stage of the procedure was assessed individually, and an evaluation was conducted on the overall effectiveness of the LA techniques.

It may be argued that the Wong–Baker Scale (WBS) serves as a more appropriate instrument for assessing the effectiveness of local anesthesia since this index is a child-oriented response to pain; however, the design of the study evaluated multiple steps during the procedures. It would not be practical to ask the patient after each step for feedback; therefore, the SEM serves as a better tool for our study, which has been validated for evaluating the effectiveness of local anesthesia. Future research may utilize the WBS as a supplementary instrument in conjunction with SEM to provide more comprehensive and pertinent findings.

The clinical relevance of our study lies in the effectiveness of IOT using QS5, which provided reliable pain control for pediatric patients undergoing pulpotomies and SSC procedures. Additionally, the study evaluated postoperative complications and found them to be less common with the IOT using QS5 compared to BIT, further supporting its effectiveness and safety in pediatric dental procedures.

## 5. Conclusions

In this study, LA administered using the QS5 system was found to be comparable to that administered via BIT for pulpotomy and SSC procedures performed on pediatric mandibular primary molars when 4% articaine and 1:100,000 epinephrine were used. However, IOT given via QS5 may be a desirable option for pediatric dentistry due to its reduced risk of postoperative complications in soft tissues.

## Figures and Tables

**Table 1:** Clinical and demographic characteristics and clinical variables of the participants (*n* = 33).

Characteristics	*n* (%)
Age (years)	
4–6	20 (60.6)
7–9	13 (39.4)
Gender	
Male	13 (39.4)
Female	20 (60.6)
Tooth type	
Lower D	13 (39.4)
Lower E	20 (60.6)
No. of injections	
Buccal infiltration	
1	27 (81.8)
>1	6 (18.2)
Intraosseous anesthesia technique	
1	24 (72.7)
>1	9 (27.3)

**Table 2:** Number of injections administered for each technique (*n* = 33).

Number of Injections	Type of Anesthesia Technique		*p*-Value
	BIT	IOT	
1	27	24	0.378
>1	6	9	

BIT: traditional buccal infiltration technique. IOT: intraosseous technique.

**Table 3:** Comparison of the mean Sounds, Eyes, and Motor (SEM) scale scores associated with the two anesthesia techniques recorded during the seven stages of the treatment procedure.

Stage of Procedure	Anesthesia Technique		Mean Difference	*p*-Value
	Buccal Infiltration	Intraosseous Anesthesia Technique		
Pulpotomy				
Rubber dam placement	3.76 (1.7)	4.00 (2.1)	−0.242	0.604
Drilling	3.82 (2.0)	3.82 (2.0)	0	0
Opening the pulp	4.0 (2.2)	4.0 (2.2)	0	0
Removal of pulpal tissue	3.76 (2.0)	3.76 (2.0)	0	0
Removal of rubber dam	3.15 (0.9)	3.33 (1.6)	−0.182	0.553
Total	18.48 (6.1)	18.91 (8.9)	−0.424	0.556
Stainless steel crown procedure				
Preparing the tooth	4.33 (1.9)	4.39 (2.3)	−0.061	0.888
Placement of the crown	3.97 (1.7)	4.03 (2.2)	−0.061	0.885
Total	8.30 (3.3)	8.42 (4.4)	−0.121	0.875

## Data Availability

The raw data supporting the conclusions of this article will be made available by the authors on request. The data are not publicly available, as the data may be subject to restrictions, including compliance with ethical guidelines or privacy concerns. Requests will be evaluated on a case-by-case basis to ensure appropriate use and adherence to any applicable regulations.
